# *Trixis angustifolia* DC. as a potential plant for the co-management of diabetes mellitus and tuberculosis

**DOI:** 10.1371/journal.pone.0339176

**Published:** 2025-12-31

**Authors:** Anuar Salazar-Gómez, Gustavo G. Flores-Bernal, Fernando Uriel Rojas-Rojas, Omar Merino-Pérez, Lucero Catalán-Gonzalez, Julieta Luna-Herrera, Leticia Garduño-Siciliano, M. Elena Vargas-Díaz

**Affiliations:** 1 Departamento de Química Orgánica, Escuela Nacional de Ciencias Biológicas, Instituto Politécnico Nacional, Ciudad de México, México; 2 Escuela Nacional de Estudios Superiores Unidad León, Universidad Nacional Autónoma de México, León, Guanajuato, México; 3 Laboratorio de Ciencias AgroGenómicas, Escuela Nacional de Estudios Superiores Unidad León, Universidad Nacional Autónoma de México, León, Guanajuato, México; 4 Departamento de Inmunología, Escuela Nacional de Ciencias Biológicas, Instituto Politécnico Nacional, Ciudad de México, México; 5 Departamento de Farmacia, Escuela Nacional de Ciencias Biológicas. Instituto Politécnico Nacional, Ciudad de México, México; University of Buea, CAMEROON

## Abstract

Diabetes mellitus and tuberculosis represent two concurrent conditions that have posed significant challenges to global public health. A comprehensive investigation into new strategies for co-managing both conditions is essential, given the possible drug interactions. *Trixis angustifolia* DC. is a plant commonly used in Mexican traditional medicine and has potential antimycobacterial and hypoglycemic properties. This study aimed to investigate the chemical composition of the ethyl acetate extract of *T. angustifolia* (TxAcE) and its antimycobacterial, hypoglycemic, and hypolipidemic activities. The structures of the isolated compounds were determined by NMR and mass spectrometry. The *in vitro* antimycobacterial activity of TxAcE and isolated compounds was determined by the Microplate Alamar Blue Assay. The hypoglycemic and hypolipidemic activities of TxAcE (50, 100, and 200 mg/kg p.o.) were investigated using alloxan-induced diabetes and Triton WR-1339-induced hyperlipidemia models in mice, respectively. The TxAcE and the mixture of two new trixanolides (**1a** and **1a’**) isolated from *T. angustifolia* exhibited antibacterial activity against *M. tuberculosis* H37Rv, showing a minimum inhibitory concentration of 6.25 µg/mL. In diabetic mice treated with TxAcE (200 mg/kg) for 15 days, non-fasting blood glucose and LDL-C levels were significantly reduced, while HDL-C levels were increased. Treatment with 50 and 100 mg/kg of the TxAcE reduced TG, VLDL-C, and LDL-C levels and increased HDL-C levels in Triton WR-1339-induced hyperlipidemic mice. These findings suggest that *T. angustifolia* is a promising source of natural antimycobacterial agents. Combined with its hypoglycemic and hypolipidemic effects, this plant has the potential to be valuable for future research in co-managing both diabetes mellitus and tuberculosis.

## Introduction

Diabetes mellitus (DM) remains one of the deadliest non-communicable diseases worldwide, with an estimated mortality of 3.4 million people in 2024 [1]. DM is a heterogeneous metabolic disorder characterized by hyperglycemia resulting from defects in insulin secretion and/or insulin action [2]. Hyperglycemia is associated with both chronic inflammatory processes and increased susceptibility to infections. People with uncontrolled DM are associated with alterations in the immune system that increase the risk of developing infectious diseases such as active tuberculosis (TB) caused by *Mycobacterium tuberculosis* [3,4]. In 2022, it was estimated that 0.37 million TB cases globally were reported in people living with DM [5]. Pharmacological management of hyperglycemia could improve TB treatment in DM patients and prevent many complications associated with both co-morbidity. However, drug-drug interactions and adverse drug reactions represent a common clinical problem during the co-management of DM and TB [6,7]. Several studies based on ethnomedicine have shown that some medicinal plants have antimycobacterial and antidiabetic effects [8–11]. Thus, preclinical investigations of medicinal plants with dual antimycobacterial and antidiabetic effects could provide valuable information for the co-management of DM and TB.

The genus *Trixis* P. Browne (Asteraceae: Mutisieae) comprises approximately 45 species [12,13], 19 of which occur exclusively in Mexico [14,15]. Many species have been traditionally used in America to treat lung diseases, diabetes, stomach illnesses, rheumatism, wounds, venereal diseases, and to improve blood circulation [16–19]. *Trixis* plants have attracted the attention of scientists for a long time due to their pharmacological potential [18,20–27] and Trixanolide constituents. Trixanolides, or trixikingolides, are structurally diverse molecules based on a trixane skeleton, which are characteristic secondary metabolites of the genus *Trixis*. Over 50 trixanolides with various structural patterns have been isolated from *Trixis* plants [22,26,28–32]. These compounds are interesting constituents that have been widely studied in the *Trixis* genus. However, few studies have been conducted to identify the biological activities of these compounds. So far, the findings have included antileishmanial and trypanocidal activities [22,26].

*Trixis angustifolia* DC. (Asteraceae), commonly known as “Hierba del viento,” “Capitana,” or “Montezuma,” is a shrub that grows to a height of 1 to 1.5 m, with leaves that are narrowly lanceolate to nearly linear and yellow flower heads [33]. It is an endemic plant of Mexico, distributed in the central and northern parts of the country [14], and is used in traditional medicine to treat lung diseases, rheumatism, wounds, and fever [34,35]. Recently, we reported the hypoglycemic and hypolipidemic effects of the aqueous extract from the aerial parts of *T. angustifolia* [24,25] and the antimycobacterial [23] and antinociceptive effects of organic extracts [27]. The main components of *T. angustifolia* are flavones, with 5,6-dihydroxy-7,8,4’-trimethoxyflavone being the most representative. Regarding trixanolides, no scientific reports have been published on their isolation or identification from this species. Thus, in line with our biological and chemical investigations of *T. angustifolia*, the present work aimed to investigate the antimycobacterial, hypoglycemic, and hypolipidemic activities of the ethyl acetate extract of *T. angustifolia* (TxAcE), as well as its chemical composition, with a focus on the isolation of trixanolides. This research provides a scientific basis for future studies on the pharmacological properties of *T. angustifolia* for co-managing DM and TB.

## Materials and methods

### Plant material

The plant material used in this study was the same specimen previously collected and documented in our earlier research [23–25,27]. *Trixis angustifolia* DC. was collected in Durango, Mexico (24°00’10.9“N 103°57’32.3”W) in April 2015. The plant material was authenticated by Professor Manuel Quintos-Escalante, and a specimen was deposited in the Herbarium of the Escuela Nacional de Ciencias Biológicas, Instituto Politécnico Nacional, with reference code: Col. M. González y S. Acevedo 2144. The plant was dried at room temperature.

### General experimental procedures

^1^H and ^13^C NMR spectra were obtained on a Varian NMR System spectrometer at 500 and 125 MHz, respectively. Chemical shifts (δ) are reported in parts per million (ppm) relative to internal tetramethylsilane (Me_4_Si, δ0.0) for ^1^H NMR and CDCl_3_ (δ77.0) for ^13^C NMR. Coupling constants (*J*) are reported in Hertz (Hz). Multiplicities are indicated by s (singlet), d (doublet), dd (doublet of doublets), ddt (doublet of doublet of triplets), t (triplet), td (triplet of doublets), m (multiplet), ovlp (overlapped with other signals). Peak assignments of the ^1^H and ^13^C NMR spectra were confirmed using 2D NMR experiments. HRMS were recorded on a micrOTOF-Q II™ spectrometer (Bruker Daltonics, Billerica, MA, United States) using desorption electrospray ionization-MS in positive mode. All solvents used for extraction and chromatography were purchased from Sigma-Aldrich and distilled before use. Analytical thin-layer chromatography (TLC) was performed using precoated TLC plates with Silica Gel 60-F254 and visualized using combinations of UV and cerium molybdate (Hanessian’s stain). Purification of compounds was performed by column chromatography (CC) on silica gel (Merck 230–400 mesh).

### Extraction and isolation

The air-dried powdered aerial parts of *T. angustifolia* (500 g) were macerated with ethyl acetate (x2) at room temperature for seven days. The resulting extracts were combined, filtered, and evaporated under reduced pressure to yield 24.9 g (4.98%) of residue. Trixanolides were extracted according to the procedure described previously [32], with some modifications. A portion of the crude extract (6 g) was suspended in EtOH (50 ml) at 60 °C, diluted with 50 ml of H_2_O, and partitioned successively with hexane and CH_2_Cl_2_ to afford a CH_2_Cl_2_ fraction (2.192 g). The CH_2_Cl_2_ fraction was subjected to CC and eluted with a hexane/EtOAc mixture of increasing polarity from hexane/EtOAc (8:2) to hexane/EtOAc (2:8). Three hundred forty-eight fractions were collected and then grouped into twenty major fractions (A-T) after TLC analysis. The regrouped fraction labeled FQ (187 mg) was redissolved in CH_2_Cl_2_/hexane and crystallized at room temperature to afford a mixture of the trixanolides **1a** and **1a’** as a white amorphous powder (19 mg); HRMS m/z 485.2132 [M + Na]^+^ (calcd. for C_25_H_34_O_8_Na, 485.2151).

### Antimycobacterial activity

#### Mycobacterial species used and inoculum preparation.

*Mycobacterium tubercul*osis H37Rv (ATCC 27294) and the nontuberculous mycobacteria *Mycobacterium smegmatis* mc2 155 (ATCC 700084) were acquired from the American Type Culture Collection. *Mycobacterium abscessus* smooth (Sm) and rough (Rm) variants were clinical isolates recovered from Mexican patients and identified by conventional microbiological methods.

*M. tuberculosis* H37Rv was grown in Middlebrook 7H9 broth enriched with 0.2% glycerol and 10% OADC (oleic acid, albumin, dextrose, and catalase) at 37ºC until the log phase growth was reached (2 weeks). Nontuberculous strains were grown in the same conditions in non-enriched Middlebrook 7H9, reaching their log phase growth in 4–7 days. All cultures were adjusted to McFarland No. 1 Tube and diluted 1:10 using the same culture media.

#### Microplate alamar blue assay.

The antimycobacterial activity of TxAcE and a mixture of **1a** and **1a’** (1:1) was tested by the Microplate Alamar Blue Assay (MABA) [37] and reported as Minimum Inhibitory Concentration (MIC) values. TxAcE and a mixture of **1a** and **1a’** (1:1) were dissolved in dimethylsulfoxide.

The MABA was performed in 96-well plates in a final volume of 200 µl containing 100 µl of the corresponding culture media and TxAcE or a mixture of **1a** and **1a’** (1:1) in concentrations ranging from 200–3.125 µg/mL and 100 µl of each bacterial suspension. The plates were incubated for 2 days for nontuberculous mycobacteria and 5 days for *M. tuberculosis* H37Rv at 37ºC. Afterward, 20 µl of Alamar blue solution were added to each well, and plates were placed at 37 ºC for 24 h. Bacterial growth was measured by the relative fluorescence units determined in a plate fluorometer (Thermo Labsystems Fluoroskan Ascent FL) at 544 nm excitation and 590 nm emission. The relative fluorescence units obtained from the wells containing only bacterial suspension were considered 100% bacterial growth control.

### Experimental animals and ethical considerations

Healthy ICR male and female mice (24 ± 3 g, aged 6–7 weeks) were obtained from PROPECUA, S.A. de C.V., Mexico City. All animals were acclimatized for seven days and maintained under standard laboratory conditions (12-h light/dark cycle, 25 ± 2 °C, and relative humidity of 55–80%), with standard diet and purified water provided *ad libitum*. All animals involved in this study were treated humanely in accordance with the recommendations in the Guide for the Care and Use of Laboratory Animals of the National Institutes of Health and the recommendations of the Mexican Official Norm for Animal Care and Handling (SAGARPA, NOM-062-ZOO-1999) [36]. The health status of the animals was regularly monitored, and every effort was made to minimize animal suffering during the study period. The protocol was approved by the Bioethics Committee of the National School of Biological Sciences of the National Polytechnic Institute (ENCB-IPN), registration number ENCB/CEI/007/2018 CONBIOETICA09CEI03720130520.

For the acute toxicity study, euthanasia criteria were set for animals experiencing severe health decline or uncontrollable distress, including significant weight loss, arching, cyanosis, hypothermia, difficulty moving, difficulty breathing, convulsions, marked behavior changes, fainting, or irreversible organ dysfunction. Any animal showing persistent signs of severe distress or pain would have been euthanized by cervical dislocation in full accordance with ethical guidelines. Since no signs of toxicity were observed in any of the animals, euthanasia was performed at the conclusion of the studies.

For blood collection, a retro-orbital puncture technique was executed under anesthesia via intraperitoneal injection (i.p.) of 1% sodium pentobarbital (50 mg/kg of body weight). At the end of the study period (immediately after blood collection), the animals were promptly and humanely euthanized by cervical dislocation in full compliance with ethical guidelines. The procedure was performed by trained personnel with technical precision to ensure rapid and effective dislocation induction, thereby minimizing pain or distress. Carcasses were disposed of safely and in accordance with established procedures.

### Acute toxicity of TxAcE

Acute toxicity was carried out according to Guideline No. 423 provided by the Organisation of Economic Co-operation and Development (OECD), with minor modifications [38]. This procedure minimizes the number of animals required for acute toxicity testing. Three female (nulliparous and non-pregnant) or three male mice were randomly assigned to each of the two experimental groups. The TxAcE, dissolved in a vehicle (Tween 80-H_2_O, 0.5%), was administered intragastrically after a 12-h fasting period at a dose of 2000 mg/kg of body weight (bw). The control group received only the vehicle (Tween 80-H_2_O, 0.5%). All animals were monitored for the next four hours after administration, then every 24 hours for 14 days for any signs of toxicity or mortality. Observations included changes in weight, skin, fur, eyes, and mucous membranes; effects on the respiratory, circulatory, autonomic, and central nervous systems, as well as on somatic motor activity and behavior patterns. Particular attention was placed on observing tremors, convulsions, salivation, diarrhea, lethargy, sleep, and coma. At the end of the experiment (14 days), all the animals were euthanized by cervical dislocation, in full compliance with ethical guidelines. Macroscopic examination was then performed on the heart, liver, spleen, lung, and kidney.

### Hypoglycemic effect of TxAcE

Male mice (24 ± 3 g) were fasted for 18 h, and a hyperglycemic condition was induced by a single injection of alloxan monohydrate (200 mg/kg, i.p.) [39]. After five days, hyperglycemia (fasting blood glucose levels greater than 400 mg/dL) was confirmed using the OneTouch Ultra 2 blood glucose meter (Johnson and Johnson, USA). The blood sampling was done by cleaning the tail with 70% ethanol and then nipping the tip of the tail. A drop of blood was placed on a blood glucose test strip and inserted into a blood glucose meter. A total of 35 mice (28 diabetic mice and seven normoglycemic mice) were used for the experiment. The diabetic mice were randomly divided into four groups (n = 7) and administered intragastrically daily with TxAcE (50, 100, and 200 mg/kg bw) for 15 days. Normoglycemic mice were administered intragastrically daily with vehicle (Tween 80-H_2_O, 0.5%). The selection of these extract doses was based on findings from our previous results [24,25]. Control group animals were also treated with the vehicle. Non-fasting blood glucose levels were monitored before daily treatments on days 3, 5, 7, 9, 11, and 15 to assess the anti-diabetic effect. At the end of the experiment (16^th^ day), the animals were fasted overnight, and blood samples were drawn by puncturing the retro-orbital sinus under anesthesia with sodium pentobarbital. Dry tubes containing collected blood were centrifuged at 13,000 × g for 15 minutes to obtain serum, which was stored at −20°C until the measurement of biochemical parameters.

### Hypolipidemic effect of TxAcE

Triton WR-1339-induced hyperlipidemia was carried out as described previously [40]. Overnight fasted mice were randomly divided into five groups of seven animals each and treated as follows: Group I served as a control (vehicle; Tween 80-H_2_O, 0.5%). Hyperlipidemia was induced in Groups II to V by a single intraperitoneal injection of Triton WR-1339 (400 mg/kg bw) dissolved in normal saline (pH 7.4). Groups III to V were administered intragastrically with TxAcE (50, 100, and 200 mg/kg bw, respectively) 1 hour before and 22 and 48 hours after the Triton WR-1339 injection. The selection of these extract doses was based on findings from our previous results [24,25]. The second group (hyperlipidemic control) was administered intragastrically with Tween 80-H_2_O, 0.5%. At the end of each treatment (4 days), the animals were fasted overnight, and blood samples were drawn by puncturing the retro-orbital sinus under anesthesia with sodium pentobarbital. Dry tubes containing collected blood were centrifuged at 13,000 × g for 15 minutes to obtain serum, which was stored at −20°C until the measurement of biochemical parameters.

#### Biochemical analysis.

Serum samples were subjected to lipid analysis as described previously [41]. TC, HDL-C, and TG were quantified using the corresponding reaction kits for the automatic Vitalab selectra two instrument (Wiener Lab, Amsterdam, Netherlands). VLDL-C and LDL-C were calculated as follows [42]: VLDL-C = TG/5 and LDL-C = TC− (HDL-C+ VLDL-C).

### Statistical analysis

All data are expressed as mean ± standard error of the mean. GraphPad Prism® version 9.0.1 for Windows software (California, USA) was used for statistical analyses. The significance was evaluated using an analysis of variance (ANOVA) and Tukey’s *post hoc* multiple-comparison test. Significant differences were set at P-values less than 0.05.

## Results and discussion

### Structural characterization of isolated compounds

**Fig 1:**
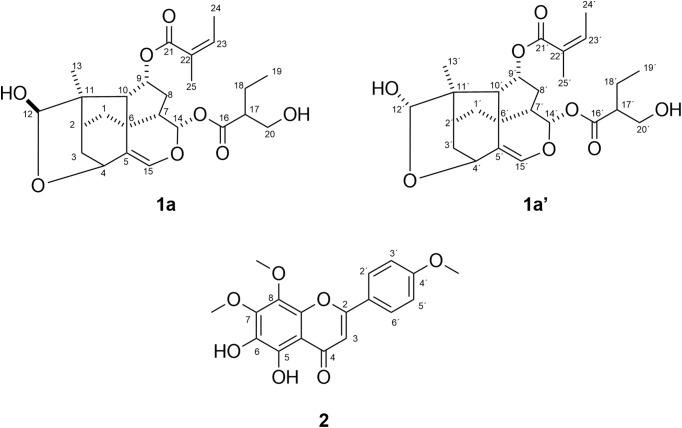
Structures of trixanolides (1a and 1a’) and flavone (2) isolated from *T. angustifolia.*

Two new trixanolides (**1a** and **1a’**) and one previously known flavone **2** were isolated from the ethyl acetate extract of *T. angustifolia* (Fig 1). The known compound, 5,6-dihydroxy-7,8,4’-trimethoxyflavone **2**, was identified by comparison of spectroscopic data with the literature [23].

Compounds **1a** and **1a’** were obtained as an inseparable mixture, appearing as a white amorphous powder. The ^1^H NMR spectrum (Table 1 and S1 Fig) of the mixture of **1a** and **1a’** showed two sets of signals in nearly 1:1 ratio, e.g., Me-13 (δ 1.23, s; 1.18, s), H-4 (δ 4.40, td, *J* = 2.8, 1.4 Hz; δ 4.31, td, *J* = 2.8, 1.3 Hz), H-12 (δ 5.01, s; δ 4.97, s) and H-15 (δ 6.32, s; δ 6.30, s). The ^13^C NMR spectrum showed 49 carbon signals, most occurring pairwise (Table 1 and S2 Fig). The ESI mass spectrum (S8 Fig) of the mixture of **1a** and **1a’** showed the sodium adduct ion peak [M + Na]^+^ at m/z 485.2132 (calcd. for C_25_H_34_O_8_Na, 485.2151), corresponding to the two isomers with the same molecular formula (C_25_H_34_O_8_).

**Table 1:** ^1^H (500 MHz) and ^13^C (125 MHz) NMR data of trixanolides 1a and 1a’ in CDCl_3._

Position	1a		1a’	
	δ_C_	δ_H_ mult. (*J* in Hz)	δ_C_	δ_H_, mult. (*J* in Hz)
1*eq*	45.0	2.77, ovlp	46.2	2.81, ovlp
1*ax*		1.30, dd (10.4, 2.7)		1.38, dd (11.0, 2.2).
2	42.8	2.10, m	40.0	2.13, m
3*eq*	33.8	2.38, ovlp	34.5	2.88, ddt (13.2, 5.1, 2.6)
3*ax*		1.51, ovlp		1.48, ovlp
4	72.2	4.39, td (2.8, 1.4)	69.8	4.31, td (2.9, 1.3)
5	123.8	–	123.7	–
6	53.6	–	53.2	–
7	41.8	2.44, ovlp	41.9	2.43, ovlp
8*eq*	39.3	2.23 ddd (18.6, 16.0, 8.4)	39.1	2.23 ddd (18.6, 16.0, 8.4)
8*ax*		2.05, ovlp		1.96, ovlp
9	75.3	5.36, ddd (5.5, 5.2, 5.1)	74.7	5.24, dd (5.2, 5.3)
10	55.6	2.79, ovlp	60.4	2.41, ovlp
11	49.2	–	47.9	–
12	97.0	4.97, s	98.9	5.00, s
13	20.5	1.18, s	19.9	1.23, s
14	95.6	5.55, d (8.5)	95.6	5.53, d (8.3)
15	134.9	6.32, s	134.4	6.30, s
16	173.8	–	173.8	–
17	49.1	2.56, m	49.1	2.56, m
18	21.5	1.69, ovlp	21.5	1.61, ovlp
19	11.6	0.97, t (7.5)	11.6	0.97, t (7.5)
20a	62.6	3.82, dd (11.1, 7.8)	62.6	3.74, ddd (11.2, 4.5, 2.2)
20b		3.82, dd (11.1, 7.8)		3.74, ddd (11.2, 4.5, 2.2)
21	166.8	–	166.8	–
22	127.2	–	127.1	–
23	139.8	6.10, ovlp	139.4	6.10, ovlp
24	15.7	2.01, ovlp	15.7	2.01, ovlp
25	20.8	1.92, ovlp	20.8	1.92, ovlp

**Fig 2:**
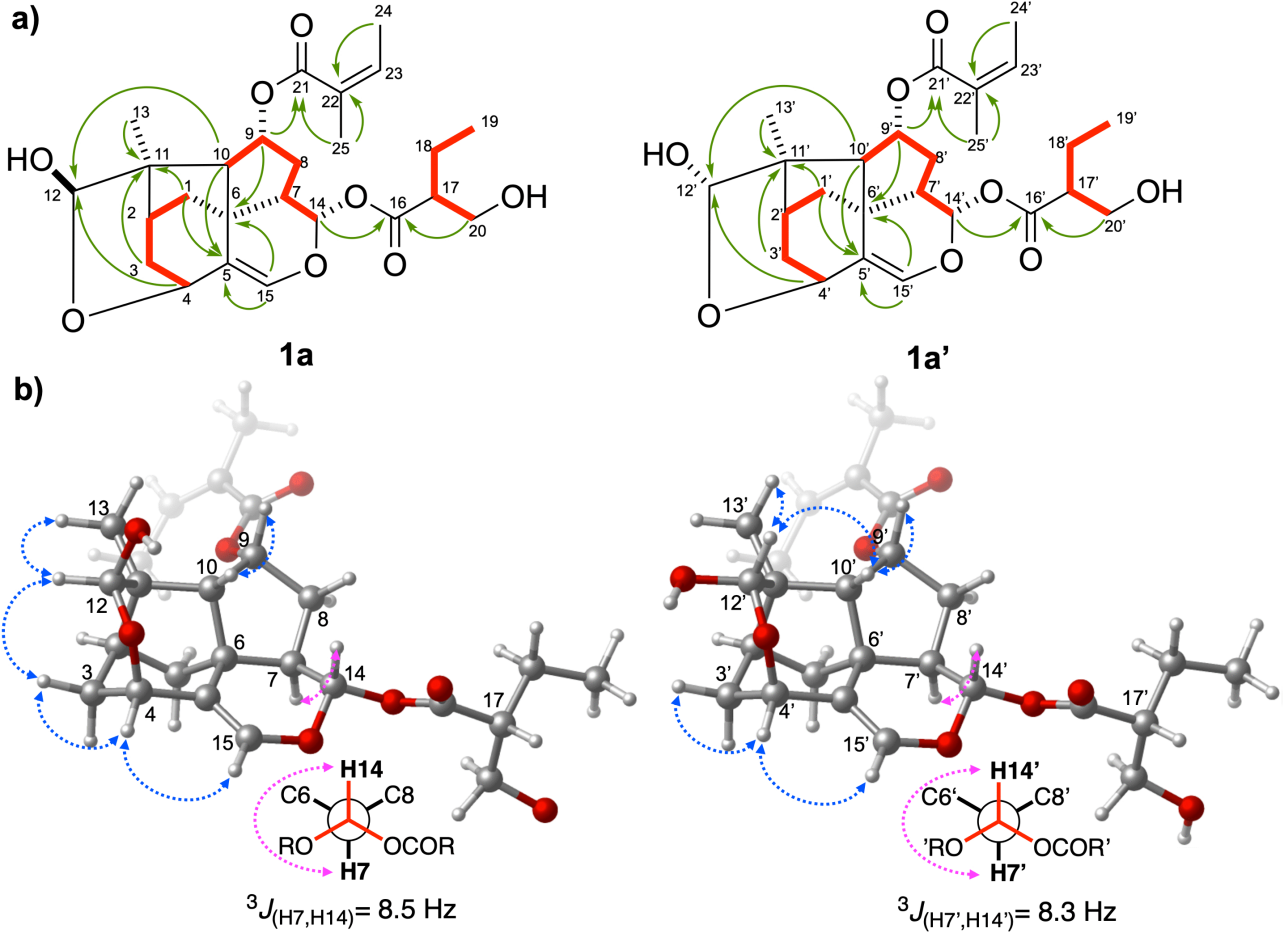
Selected a) HMBC (→), COSY (bold red line), and b) ROESY (blue ↔) correlations and ^3^*J*_(H7,H14)_ (pink ↔) of trixanolides 1a and 1a’. The configuration of C17 in the 3D models was arbitrarily assigned.

Despite the high complexity of the NMR spectroscopic data of the mixture due to the structural similarity and proximity of the chemical shifts of the two compounds, the peak assignments of the ^1^H and ^13^C NMR spectra of **1a** and **1a’** were achieved using 2D NMR experiments (COSY, TOCSY, HSQC, HMBC, and ROESY) (S3–S7 Figs) and by comparison with data in the literature [30]. NMR analyses allowed us to identify the 9,14-diester-12-hydroxytrixanolide skeleton of **1a** and **1a’**, suggesting that these compounds were analogs of four sets of C-12 epimers isolated earlier from *Trixis praestans* (Vell.) Cabrera [30]. Since one of the most remarkable changes was the chemical shifts of singlet signals at δ 5.00/4.97 for H-12, these signals were selected as the starting point for the NMR assignments of each compound. The HMBC correlations from H-9/H-15 to C-6, H-1/H-3/H-13 to C-11, H-4/H-10 to C-12, H-1/H-10/H-15 to C-5; and the corresponding 1H-1H COSY correlations from H-1, 2, 3, 9 and H-10 as well as from H-7 via H-8a,b and H-9 to H-10 confirmed the presence of the trixane core (C1-C10) (Fig 2). Unlike the 1:1 mixture of C-12 epimers of trixanolides isolated previously [30], angelate group ((2*Z*)-2-methylbut-2-enoate) was located at C-9 and the ester group 2-(hydroxymethyl) butanoate at C-14 [δ_C_ 173.8 (C-16), 49.1 (C-17), 21.5 (C-18), 11.6 (C-19), 62.5 (C-20)]. The distribution of the two acyl groups, angelate and 2-(hydroxymethyl) butanoate, over C-9 and C-14 were supported by the HMBC correlation of H-9 (δ 5.36/5.35) to C-21(δ 166.8) and H-14 (δ 5.54, 5.43) to C-16 (δ 173.8), respectively (S6 Fig). The ^13^C NMR data of the mixture showed duplicated signals for 9,14-diester-12-hydroxytrixanolide skeleton (C1-C14), while no duplication was observed for angelate and 2-(hydroxymethyl)butanoate groups. The relative configuration of **1a** and **1a’** was elucidated based on ROESY experiments, and to confirm their observed correlations, a computer-assisted 3D structure was obtained by using the software Spartan 10, V1.0.1, with MMFF94 calculations for energy minimization (Fig 2). Different ROE correlations of each H-12 signal were observed. H-12 in **1a** shows spatial coupling with H-3*eq*, while H-12’ in **1a’** is spatially coupled with H-10*’*. Based on the above, it was deduced that compounds **1a** and **1a’** correspond to the α- and β- isomers of the hydroxy group, differing in the configuration of the hemiacetalic carbon C12, similar to the anomeric carbon in carbohydrates. Additionally, several inseparable epimeric mixtures of trixanolides have been reported in previous works [31,32]. To the best of our knowledge, this is the first report of the isolation of trixanolides from *T. angustifolia*.

### Antimycobacterial activity

Traditionally, the aerial parts of *T. angustifolia* are used to treat lung diseases and fever [33,34]. These uses directed us to investigate the therapeutic potential of *T. angustifolia* for TB treatment. The results of the microplate MABA showed that TxAcE exhibited a MIC value of 6.25 µg/mL for *Mycobacterium tuberculosis* H37Rv (Table 2). We previously reported a MIC value of 50 µg/mL for an ethyl acetate extract of *T. angustifolia* obtained by consecutively extracting the aerial parts with solvents in increasing order of polarity [23]. In the present study, the plant material was subjected to direct maceration with ethyl acetate, which appears to improve the extraction of antimycobacterial compounds.

**Table 2:** Minimal inhibitory concentrations (MIC) of the ethyl acetate extract of *Trixis angustifolia* DC. (TxAcE) and trixanolides (1a and 1a’) against *Mycobacterium tuberculosis* H37Rv and nontuberculous mycobacteria.

Mycobacterium strains	MIC (μg/mL)	
	TxAcE	Mixture of 1a and 1a’ (1:1)
*M. tuberculosis* H37Rv	6.25	6.25
*M. smegmatis* mc^2^	50	ND
*M. abscessus* Rm	12.5	ND
*M. abscessus* Sm	6.25	ND

Data are means of three determinations by microplate Alamar blue assay. ND: Not determined.

The inhibitory activity of organic extracts from Asteraceae plants against *M. tuberculosis* has been previously reported [43–45], indicating the potential of this family as a source of novel compounds for TB treatment. In addition to *M. tuberculosis* infections, the prevalence of diseases caused by drug-resistant nontuberculous mycobacteria infections has increased worldwide (mainly in industrialized countries) over the past decades, some even bigger than TB prevalence [46]. Within these mycobacteria species, the *Mycobacterium abscessus* group is one of the common nontuberculous mycobacteria responsible for lung and skin infections in immunocompetent and cystic fibrosis patients [47], which has generated the medical need to discover more effective drugs against this group of mycobacteria. The TxAcE also had antimycobacterial activity against *M. abscessus* Sm (MIC = 6.25 µg/mL) and Rm (MIC = 12.5 µg/mL). These strains represent the two *M. abscessus* variants frequently recovered from distinct clinical outcomes [48]. Additionally, *M. abscessus* is considered one of the most drug-resistant mycobacterial strains [46]; thus, TxAcE could be considered a promising source of new drugs for *M. abscessus*-infection. We also test TxAcE against the strain *Mycobacterium smegmatis* mc^2^ 155. The extract showed a MIC value of 50 µg/mL, which is lower than the reported for ethyl acetate extracts from other Asteraceae plants like *Ambrosia confertiflora* DC. against *M. tuberculosis* H37Rv [44] and *Bidens odorata* (Cavanilles). J. against *M. smegmatis* mc^2^ 155 [45]. This result indicates the potential use of *M. smegmatis* mc^2^ 155 as a safe and easy-to-handle model for further studies on the mechanisms of action of *T. angustifolia* antimycobacterial compounds.

In addition to previous work by our group on the antimycobacterial activity of *T. angustifolia* hexanic extract (TxHxE) with a MIC value of 25 µg/mL [23], the activity of TxAcE described above with lower MIC values contributes to research on natural alternatives using Mexican *Trixis* species (or isolated compounds) for treating different infectious diseases caused by multiresistant bacteria. Regarding this issue, the mixture of trixanolides **1a** and **1a’** (1:1) was also evaluated for its antimycobacterial activity against *M. tuberculosis* H37Rv. Interestingly, the **1a** and **1a’** mixture exhibited significant activity against *M. tuberculosis* H37Rv with a MIC of 6.25 µg/mL (Table 2). The lower MIC reported in the present work may be due to the use of partially purified molecules rather than the mixture of molecules that composed the TxHxE.

In the previous work on the antimycobacterial activity of TxHxE, the active compounds responsible for the biological activity were not identified. In that study, flavone **2** was identified in an active fraction of TxHxE, but the isolated compound lacked antimycobacterial activity. However, the antimycobacterial activity of the TxHxE active fraction was improved after enrichment with **2**, suggesting a synergistic effect between different compounds of *T. angustifolia* [23]. To our knowledge, we report the antimycobacterial activity of trixanolides for the first time, expanding the previously reported spectrum of sesquiterpene derivatives isolated from plants against microbial pathogens [49]. This family of compounds is characteristic of the *Trixis* genus and has been the subject of several studies in different species [22,26,28–32]. Further investigations are needed to identify the structural components of trixanolides related to their activity against infectious agents and to propose them as the structural basis for developing new antimicrobial drugs.

### Acute toxicity of TxAcE

The acute toxicity results showed that neither mortality nor toxic signs were observed in animals after the TxAcE was administered at a dose of 2000 mg/kg. Additionally, no significant gross pathological changes were observed in vital organs. These results suggest that the LD_50_ of the TxAcE examined in the present study is greater than 2000 mg/kg and can be classified in class 5 according to the OECD 423 guidelines [38].

### TxAcE improves glucose and lipid profiles in diabetic mice

**Fig 3:**
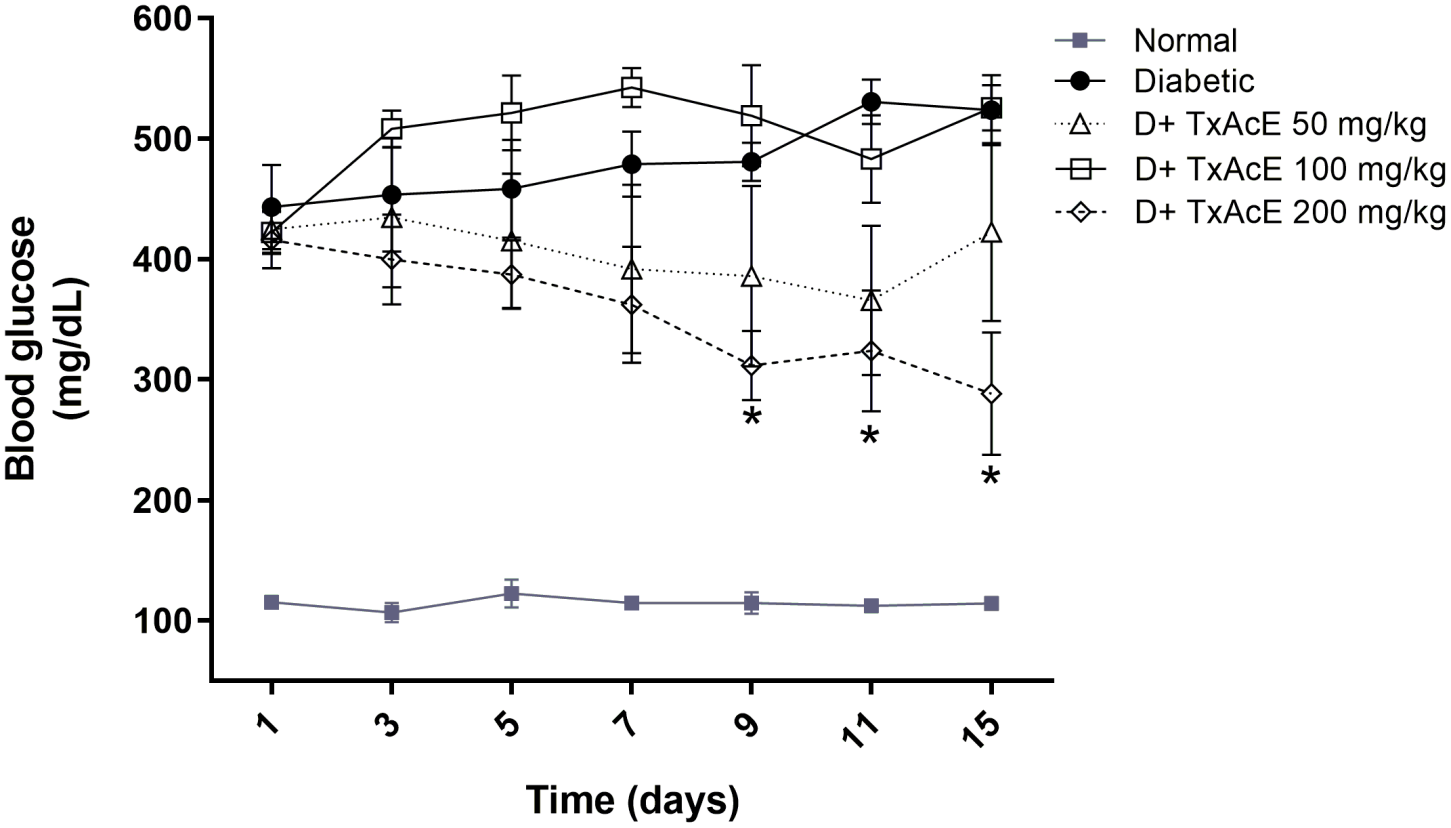
Effect of the ethyl acetate extract of *Trixis angustifolia* DC. (TxAcE) on blood glucose levels in alloxan-induced diabetic mice after 15 days of treatment. Data are expressed as mean ± SEM with n = 7. *p < 0.05 compared to the diabetic group. D: Diabetic.

DM is a medical condition characterized by hyperglycemia, defects in insulin secretion, insulin action, or both, and is associated with abnormalities in carbohydrate, protein, and lipid metabolism [50]. DM remains one of the deadliest non-communicable diseases worldwide, with an estimated mortality of 3.4 million people in 2024 [1]. Despite advances in modern medicine, medicinal plants and their derivatives are still promising options for improving treatment for diabetic patients [51]. Our previous *in vivo* study showed that an aqueous extract of *T. angustifolia* has hypoglycemic and hypolipidemic effects in alloxan-induced diabetic mice, and we found that flavone **2** is also present in the ethyl acetate fraction derived from this extract [24,25]. Motivated by these outcomes, we decided to investigate the hypoglycemic effect of TxAcE in this model. Alloxan is used as a hyperglycemic-inducing agent in experimental animals due to its selective pancreatic islet β-cell cytotoxicity, leading to hyperglycemia and diabetic complications [52]. In this study, intraperitoneal administration of alloxan to mice significantly increased blood glucose levels five days after injection. This increase persisted and progressed until the end of the experimental period (Fig 3, S1 File). However, daily treatment with TxAcE (200 mg/kg) for 15 days gradually reduced non-fasting blood glucose from 415.86 ± 23.4 mg/dL to 288.14 ± 50.8 mg/dL compared to the alloxan-induced diabetes group (523.57 ± 29.3 mg/dL). The hypoglycemic effect was observed from the 9^th^ day to the end of the study (15^th^ day), with a marked blood glucose reduction of 35.21% (*p* ≤ 0.05) and 44.97% (*p* ≤ 0.05), respectively. This reduction observed at the 15th day was even lower than the previous reduction reported (30.4%) when 100 mg/kg of the aqueous extract of *T. angustifolia* was administered in a similar time lapse experiment [25]. Treatment with a lower dose of TxAcE (50 mg/kg) in hyperglycemic mice lowered blood glucose levels on the 11^th^ day (−31.07%), but this effect was not observed until the end of the study (−19.29%). This result could be explained by the dose-response phenomenon called hormesis, which results from several factors, such as the action of a complex mixture of bioactive constituents or interactions among these compounds [53,54]

DM is associated with abnormal blood lipid metabolism, which includes elevated fasting plasma triglycerides (TG) levels, small dense low-density lipoprotein-cholesterol, and low levels of high-density lipoprotein cholesterol (HDL-C) [55]. These factors increase the risk of heart disease. Alloxan-induced diabetic mice showed a significant increase in low-density lipoprotein-cholesterol (LDL-C) levels, while HDL-C levels were significantly decreased compared to normal control mice (Table 3). The treatment with TxAcE (200 mg/kg) induced a significant reduction in LDL-C (−64.28%) and an increase in HDL-C (40.07%) levels compared with the alloxan-induced diabetic group, suggesting that the extract can improve the alloxan-induced perturbations of serum lipids in diabetic mice. The improvement of glycemia could mediate this effect, observed at a 200 mg/kg extract dose. Thus, these results indicated that *T. angustifolia* could help improve dyslipidemia and prevent certain diabetic complications.

**Table 3:** Effect of the ethyl acetate extract of *Trixis angustifolia* DC. (TxAcE) on lipid profile of alloxan-induced diabetic mice.

Group	Serum lipid profile (mg/dL)				
	TC	TG	VLDL-C	LDL-C	HDL-C
Normal	89.48 ± 5.93	86.57 ± 10.03	17.31 ± 2.01	12.51 ± 4.31	59.66 ± 3.63
Diabetic	90.59 ± 7.24	84.43 ± 8.53	16.89 ± 1.71	33.94 ± 5.93^a^	39.76 ± 3.96^a^
D + TxAcE 50 mg/kg	71.53 ± 6.79	72.26 ± 10.58	14.45 ± 2.12	13.32 ± 2.56	43.76 ± 4.01
D + TxAcE 100 mg/kg	85.06 ± 8.52	73.79 ± 14.97	14.76 ± 2.99	20.10 ± 5.64	50.21 ± 8.14
D + TxAcE 200 mg/kg	91.14 ± 4.88	98.74 ± 8.91	19.75 ± 1.78	12.12 ± 3.07^b^	59.27 ± 1.32^b^

Data expressed as a mean ± SEM; n = 7 animals/group. Diabetic (D)

^a^*p* < 0.05; the significant difference with respect to control (Normal).

^b^*p* < 0.05; the significant difference with respect to diabetic group.

### Effect of TxAcE on Triton WR-1339-induced hyperlipidemia

Dyslipidemia is common among people living with type 2 diabetes and is considered a major risk factor for cardiovascular disease. Lipid-lowering drugs are important for improving diabetic complications [56]. Therefore, the lipid-lowering activity of TxAcE was also investigated in Triton WR-1339-induced hyperlipidemia in mice. Triton WR-1339 prevents TG catabolism by inhibiting lipoprotein lipase activity, blocking the uptake of TG-rich lipoproteins from the circulation [57,58]. In this study, Triton WR-1339 (400 mg/kg) caused a significant elevation in serum levels of TG (55.23%), Very-low-density lipoprotein cholesterol (VLDL-C) (55.25%), and LDL-C (73.99%), and a marked decrease in HDL-C level (−68.37%) compared to the normal group (Table 4). After treatment with 50, 100, and 200 mg/kg of the TxAcE, a significant reduction in TG and VLDL-C levels was observed compared to the hyperlipidemic group. Additionally, serum levels of LDL-C decreased significantly at all doses (Table 4).

**Table 4:** Effect of the ethyl acetate extract of *Trixis angustifolia* DC. (TxAcE) on Triton WR-1339-Induced Hyperlipidemia.

Group	Serum lipid profile (mg/dL)				
	TC	TG	VLDL-C	LDL-C	HDL-C
Control	88.15 ± 11.18	87.82 ± 5.95	17.56 ± 1.19	10.97 ± 7.46	59.62 ± 5.88
TRW	114.91 ± 16.93	196.18 ± 21.16^a^	39.24 ± 4.23 ^a^	40.26 ± 12.47 ^a^	35.41 ± 4.65
TRW + TxAcE 50 mg/kg	97.78 ± 8.21	136.69 ± 10.21 ^b^	27.34 ± 2.04 ^b^	7.91 ± 1.96 ^b^	62.53 ± 7.57 ^b^
TRW + TxAcE 100 mg/kg	96.73 ± 6.30	122.86 ± 14.24 ^b^	24.57 ± 2.85 ^b^	4.10 ± 1.58 ^b^	68.06 ± 6.99 ^b^
TRW + TxAcE 200 mg/kg	90.05 ± 9.23	141.31 ± 9.71 ^b^	28.26 ± 1.94 ^b^	9.69 ± 2.37 ^b^	52.09 ± 7.44

Data expressed as a mean ± standard error; n = 7 animals/group. Triton WR-1339-Induced Hyperlipidemia (TRW)

^a^*p* < 0.05; the significant difference with respect to control.

^b^*p* < 0.05; the significant difference with respect to the hyperlipidemic group.

In contrast, HDL-C levels increased significantly at 50 and 100 mg/kg doses. According to a previously proposed role for HDL-C [59], the increased levels of HDL-C observed after the administration of TxAcE could contribute to cholesterol removal from peripheral tissues, resulting in the decreased levels of LDL-C observed in serum. This reduction is promising since LDL-C levels constitute the main modifiable risk factor for cardiovascular disease [60]. These observations are similar to our previous results showing a decrease of TG and VLDL-C and an increase of HDL-C in Triton WR-1339-induced hyperlipidemic mice after treatment with the aqueous extract of *T. angustifolia* [24]. Therefore, compounds with this effect could be extracted using water or ethyl acetate.

## Conclusion

Plants used in traditional medicine offer a promising source of molecules with potential applications in health care. Mexico has a high diversity of medicinal plants; among them, *T. angustifolia* is used by the Mexican population to treat different diseases. Here, we demonstrate that an ethyl acetate extract from *T. angustifolia* and a mixture of two new trixanolides (**1a** and **1a’**) isolated exhibited significant antimycobacterial activity. Furthermore, the effectiveness of this extract in reducing blood glucose and LDL-C in diabetic mice suggests its potential value for future research in co-managing both diabetes mellitus and tuberculosis. This study also contributes to the pharmacological characterization of this plant and supports its use in Mexican traditional medicine.

## Supporting information

S1 Fig ^1^H NMR spectrum of mixture of 1a and 1a’ (500 MHz, CDC_l3_). (PDF)

S2a Fig ^13^C NMR spectrum of mixture of 1a and 1a’ (125 MHz, CDC_l3_). (PDF)

S2b Fig ^13^C NMR spectrum of mixture of 1a and 1a’ (125 MHz, CDCl3). (PDF)

S3 Fig COSY spectrum of mixture of 1a and 1a’ (500 MHz, CDC_l3_). (PDF)

S4 Fig TOCSY spectrum of mixture of 1a and 1a’ (500 MHz, CDC_l3_). (PDF)

S5a Fig HSQC spectrum of mixture of 1a and 1a’ (500 MHz, CDC_l3_). (PDF)

S5b Fig HSQC spectrum of mixture of 1a and 1a’ (500 MHz, CDC_l3_). (PDF)

S6a Fig HMBC spectrum of mixture of 1a and 1a’ (500 MHz, CDC_l3_). (PDF)

S6b Fig HMBC spectrum of mixture of 1a and 1a’ (500 MHz, CDC_l3_). (PDF)

S6c Fig HMBC spectrum of mixture of 1a and 1a’ (500 MHz, CDC_l3_). (PDF)

S7 Fig ROESY spectrum of mixture of 1a and 1a’ (500 MHz, CDC_l3_). (PDF)

S8 Fig HRMS-ESI spectrum (positive ion mode) of mixture of 1a and 1a’. (PDF)

S1 File Values behind the means used to build graphs and tables. (XLSX)
