# Supplementary figures and images for: Trixis angustifolia DC. as a potential plant for the co-management of diabetes mellitus and tuberculosis
Source: PLoS One. 2025 Dec 31;20(12):e0339176. doi: 10.1371/journal.pone.0339176 (PMC12755760; doi:10.1371/journal.pone.0339176)

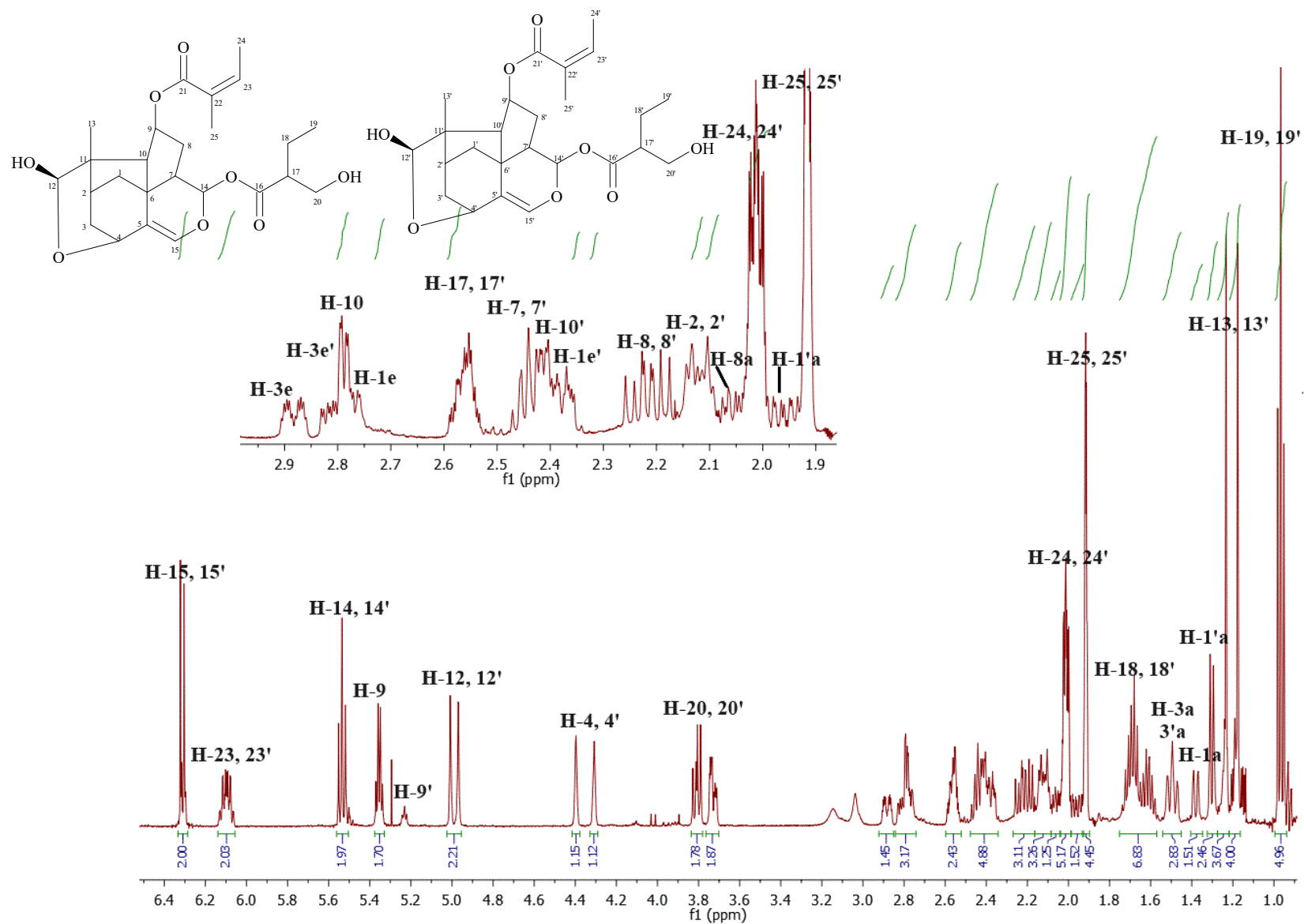

S1 Fig.  $^1\text{H}$  NMR spectrum of mixture of 1a and 1a' (500 MHz,  $\text{CDCl}_3$ ).

Supplement: S1 Fig — (PDF) [file pone.0339176.s001.pdf]

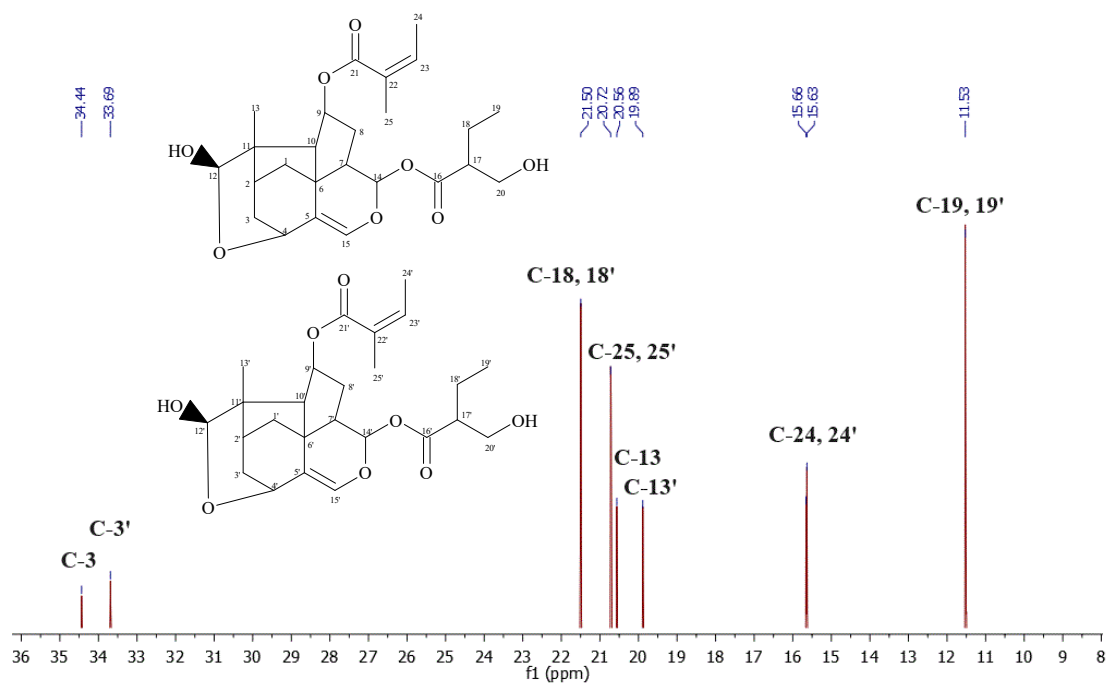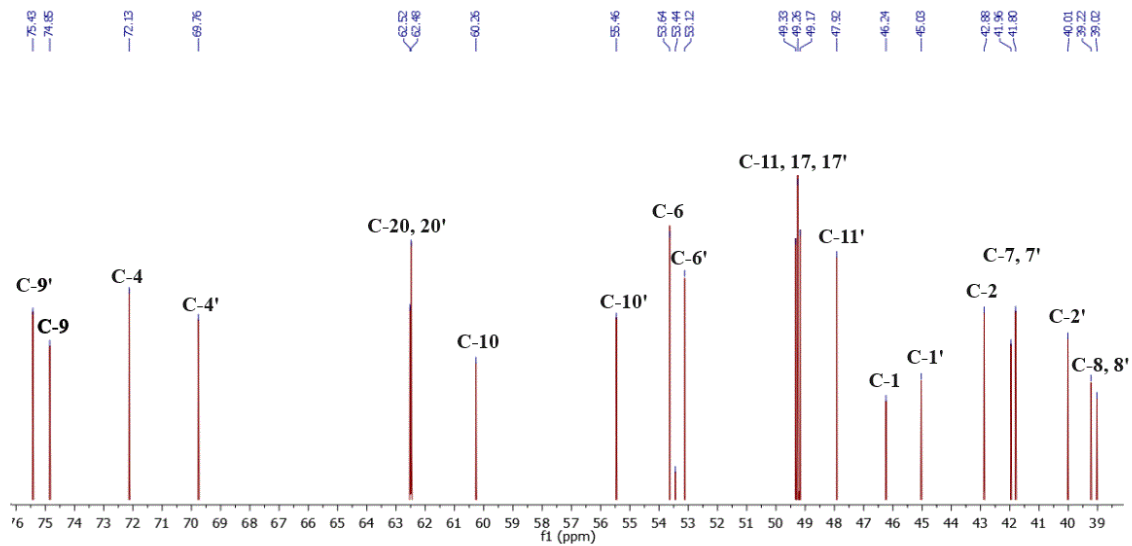

S2a Fig.  $^{13}\text{C}$  NMR spectrum of mixture of 1a and 1a' (125 MHz,  $\text{CDCl}_3$ ).

Supplement: S2a Fig — (PDF) [file pone.0339176.s002.pdf]

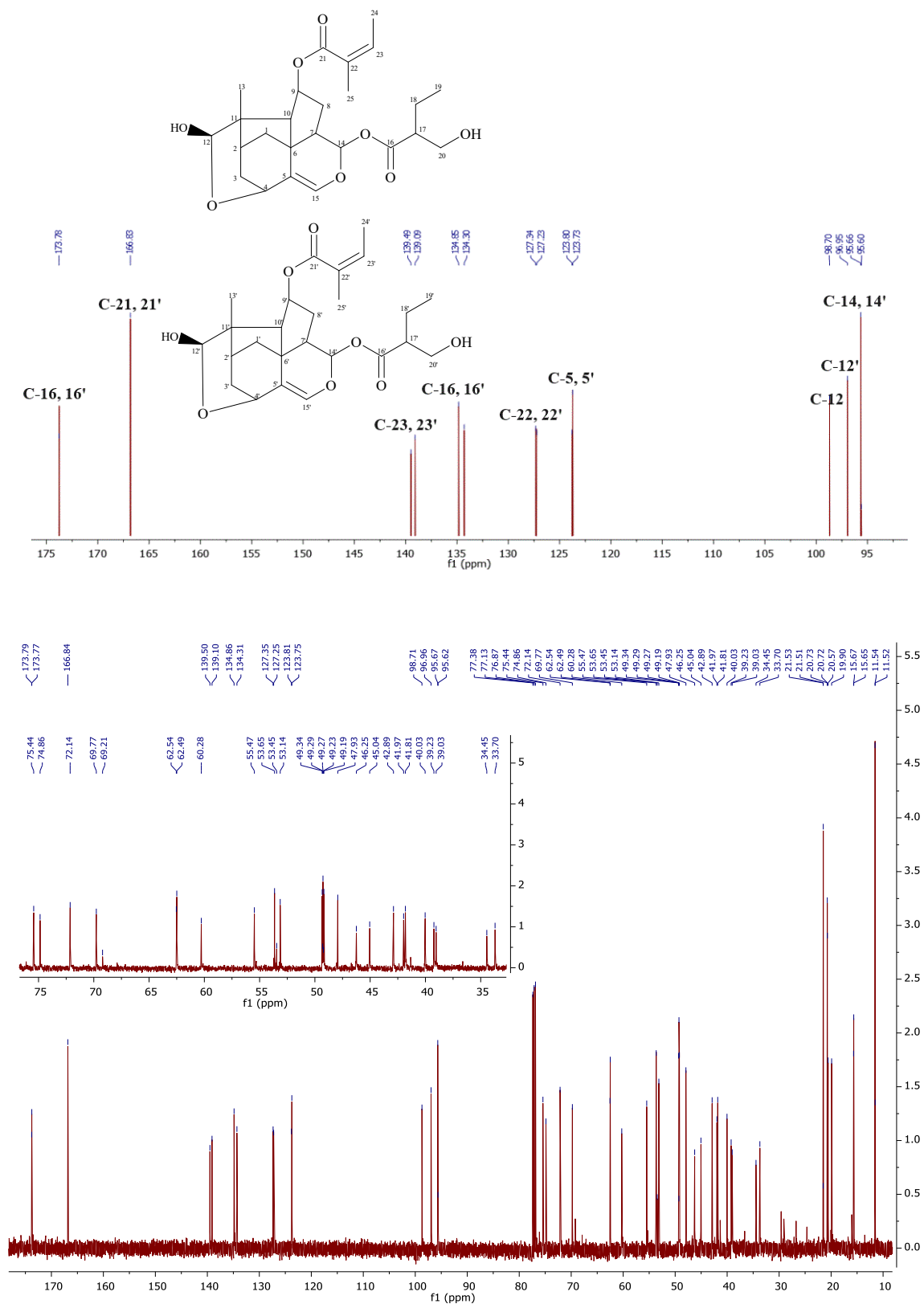

S2b Fig. <sup>13</sup>C NMR spectrum of mixture of 1a and 1a' (125 MHz, CDCl<sub>3</sub>).

Supplement: S2b Fig — (PDF) [file pone.0339176.s003.pdf]

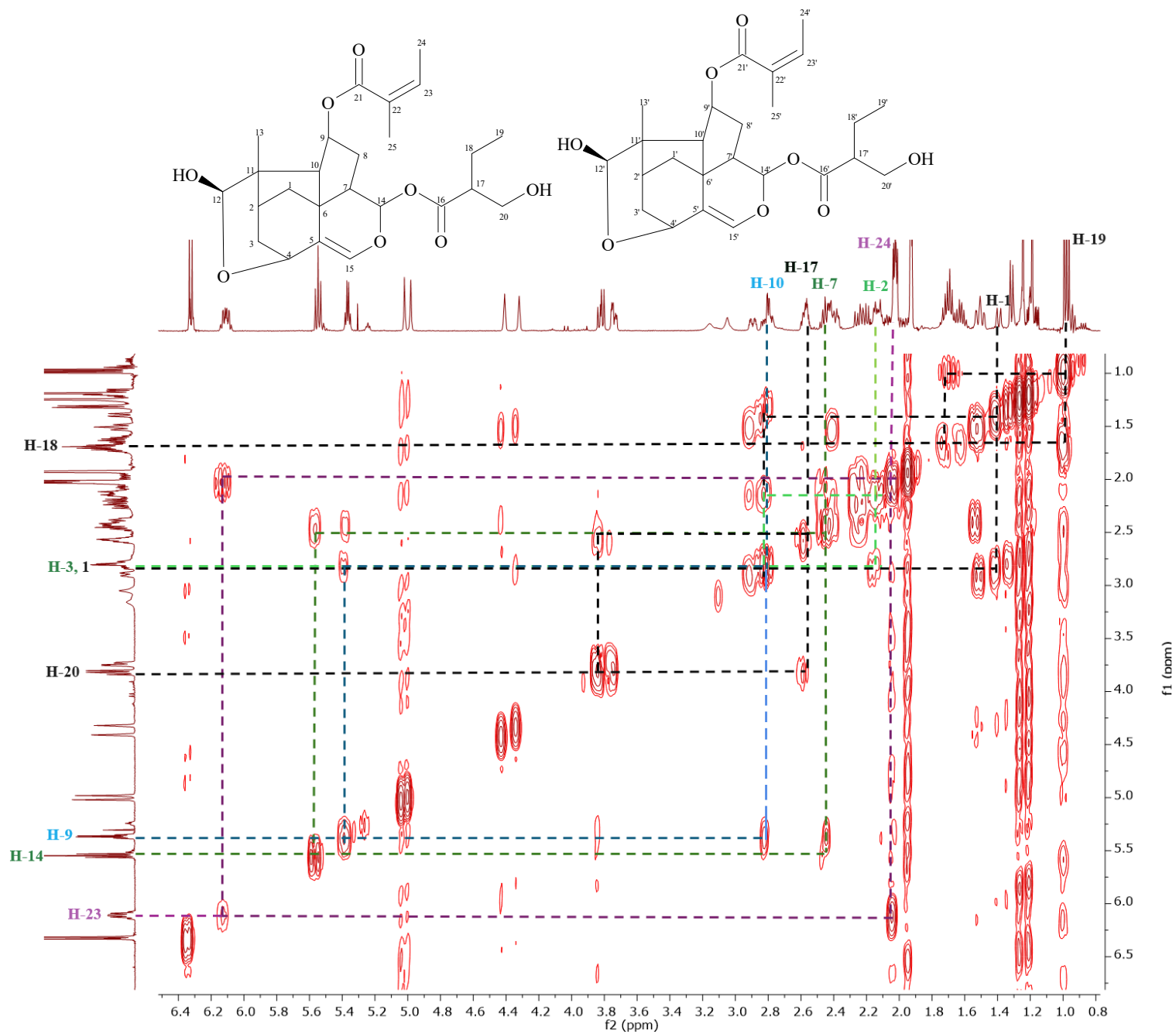

S3 Fig. COSY spectrum of mixture of 1a and 1a' (500 MHz, CDCl<sub>3</sub>).

Supplement: S3 Fig — (PDF) [file pone.0339176.s004.pdf]

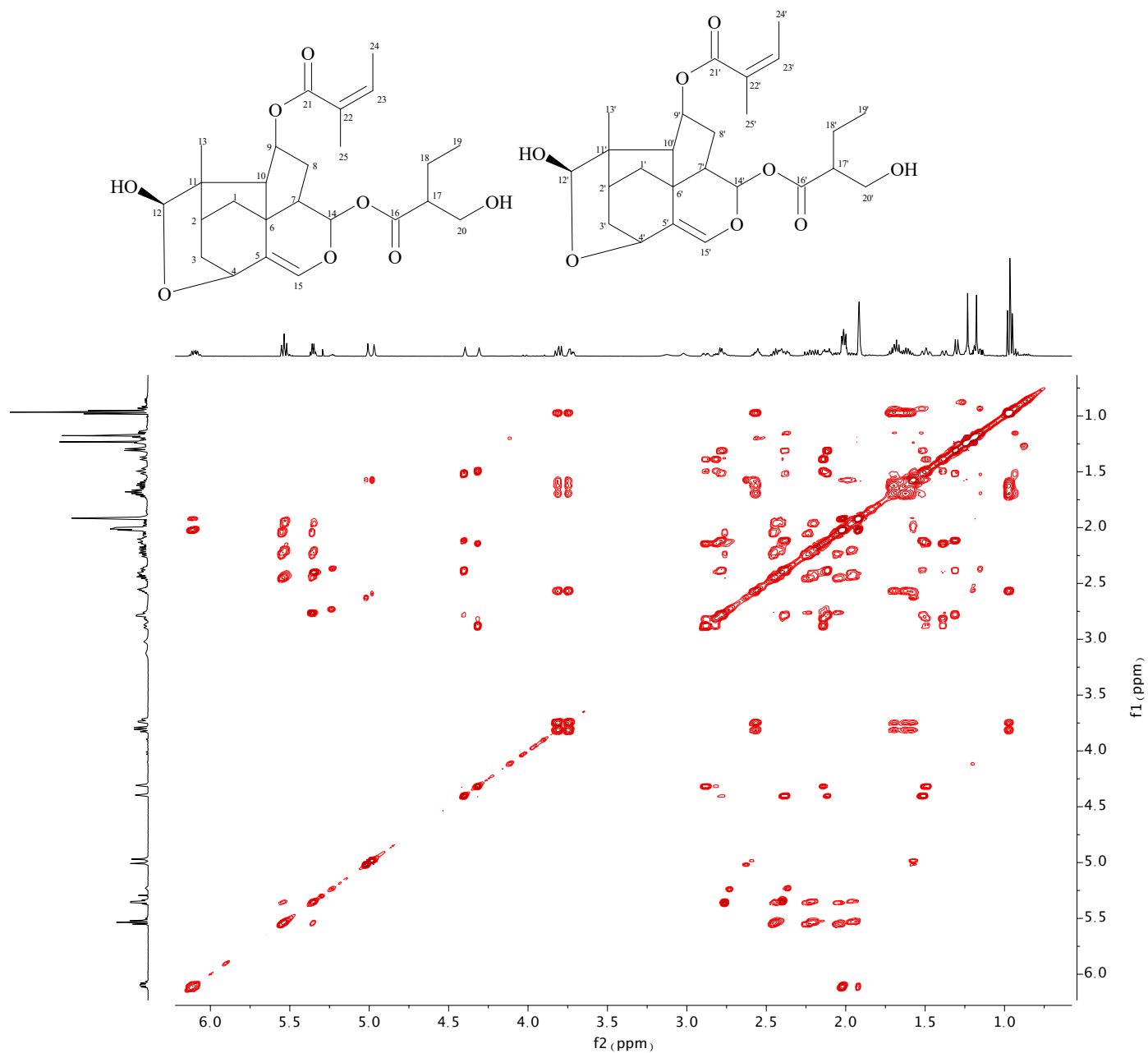

S4 Fig. TOCSY spectrum of mixture of 1a and 1a' (500 MHz, CDCl<sub>3</sub>).

Supplement: S4 Fig — (PDF) [file pone.0339176.s005.pdf]

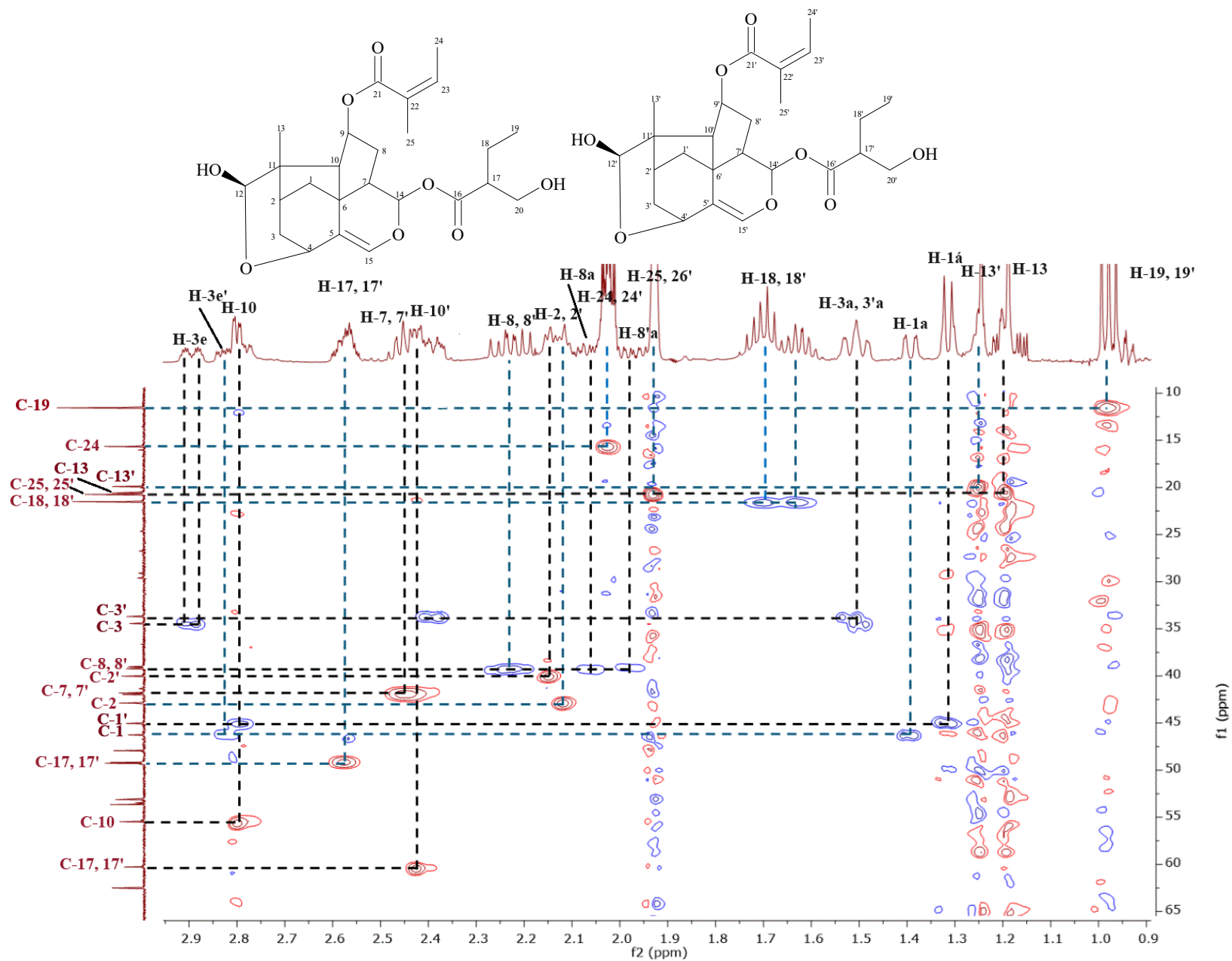

S5a Fig. HSQC spectrum of mixture of 1a and 1a' (500 MHz, CDCl<sub>3</sub>).

Supplement: S5a Fig — (PDF) [file pone.0339176.s006.pdf]

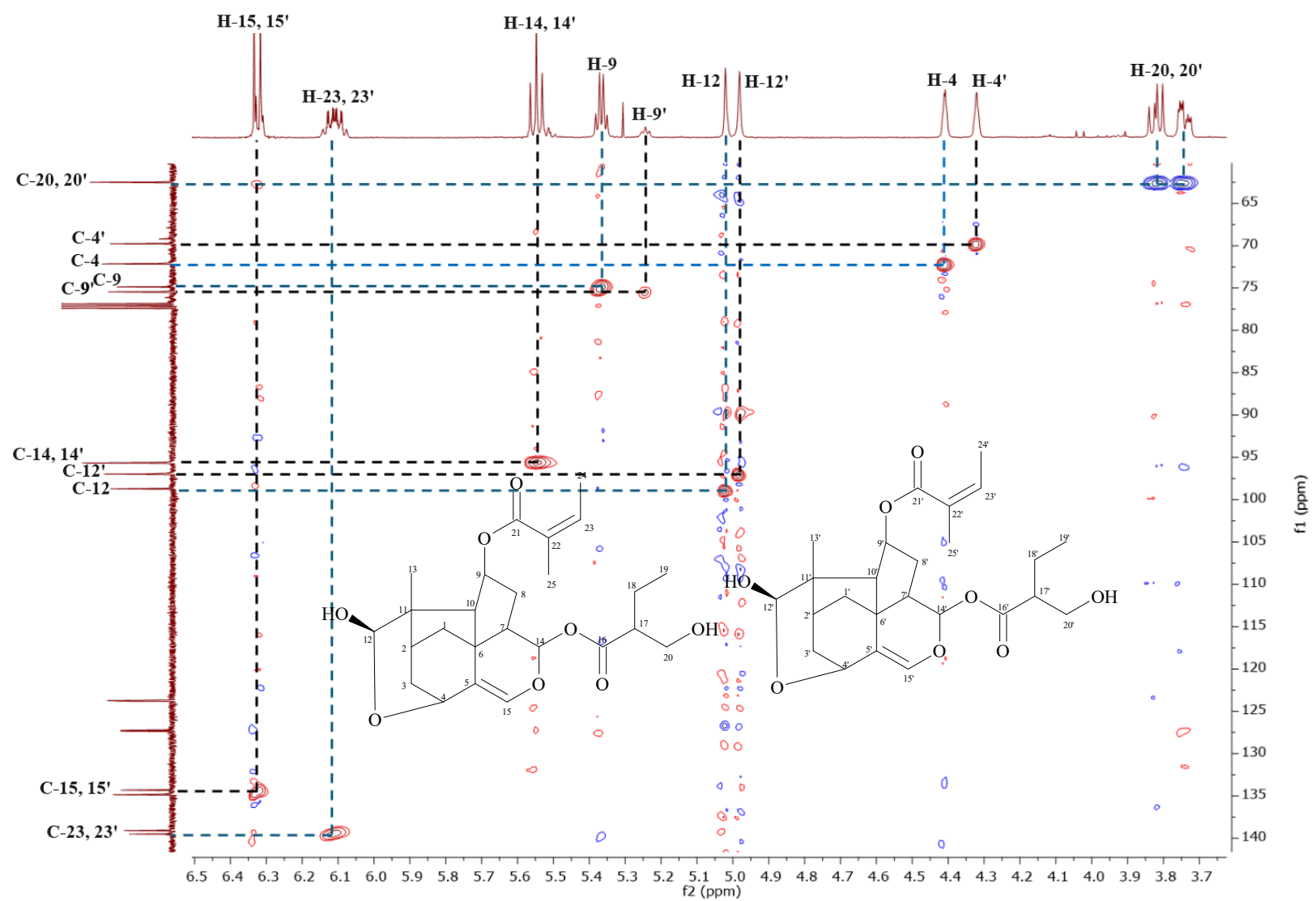

S5b Fig. HSQC spectrum of mixture of 1a and 1a' (500 MHz, CDCl<sub>3</sub>).

Supplement: S5b Fig — (PDF) [file pone.0339176.s007.pdf]

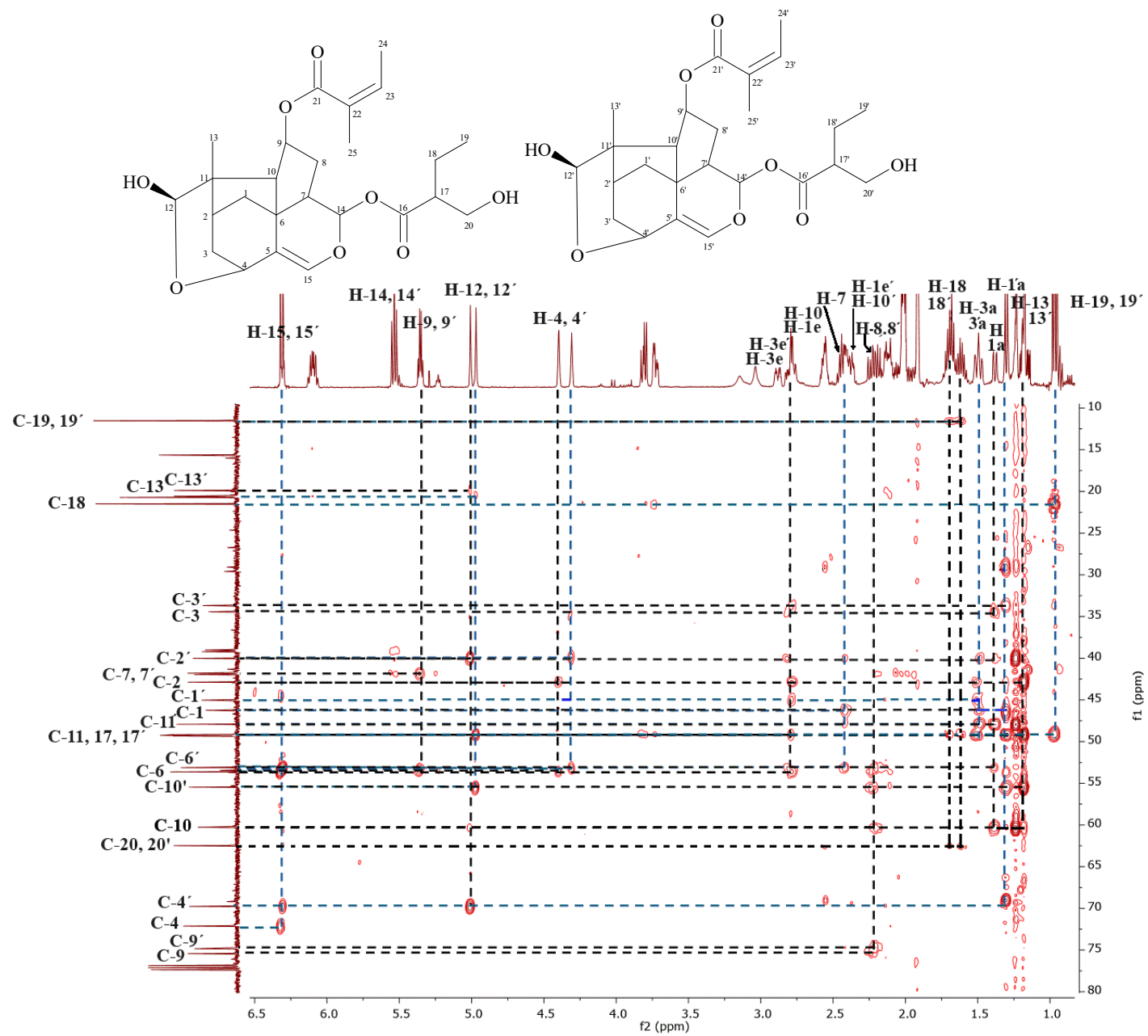

S6a Fig. HMBC spectrum of mixture of 1a and 1a' (500 MHz,  $\text{CDCl}_3$ ).

Supplement: S6a Fig — (PDF) [file pone.0339176.s008.pdf]

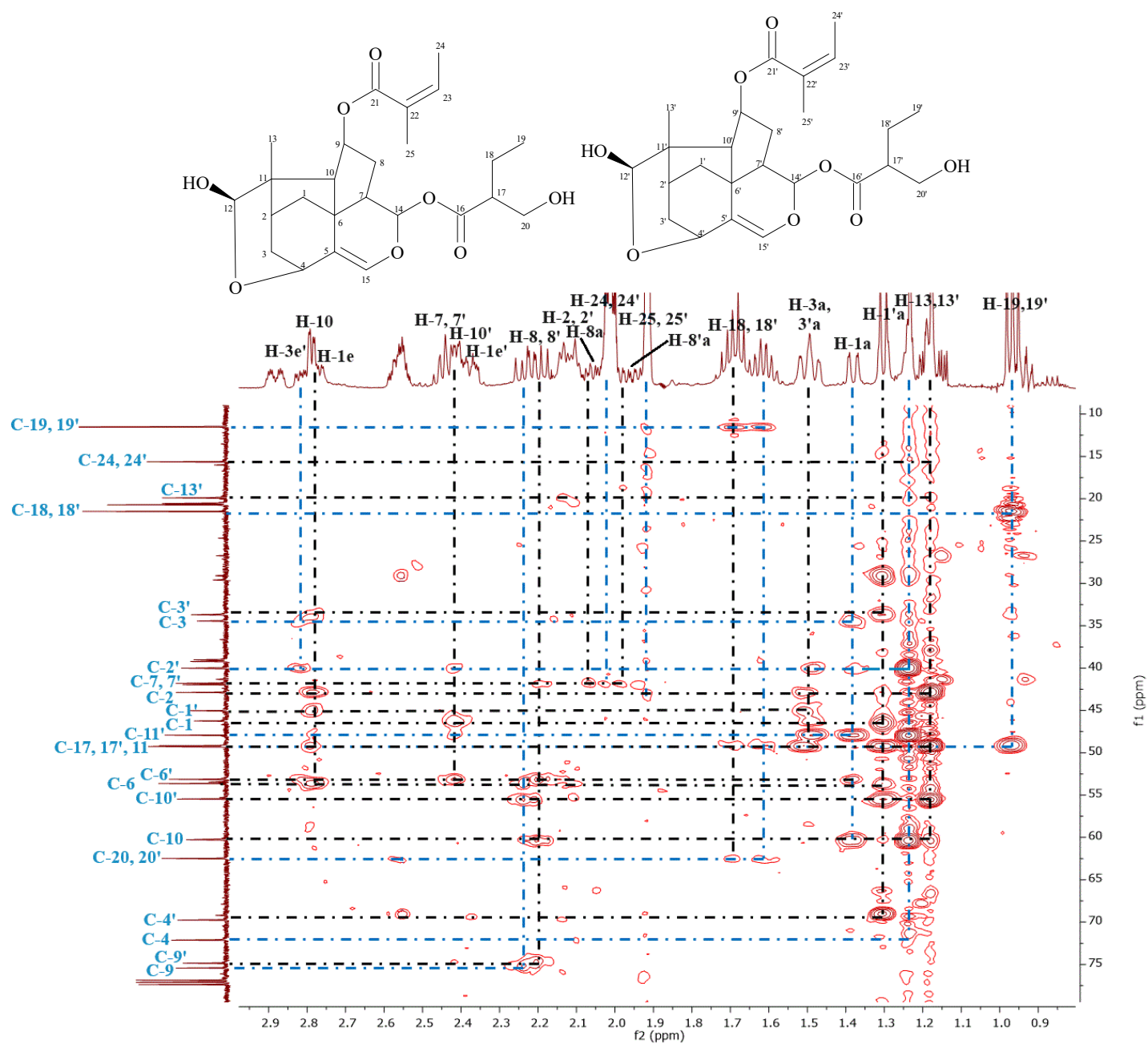

S6b Fig. HMBC spectrum of mixture of 1a and 1a' (500 MHz,  $\text{CDCl}_3$ ).

Supplement: S6b Fig — (PDF) [file pone.0339176.s009.pdf]

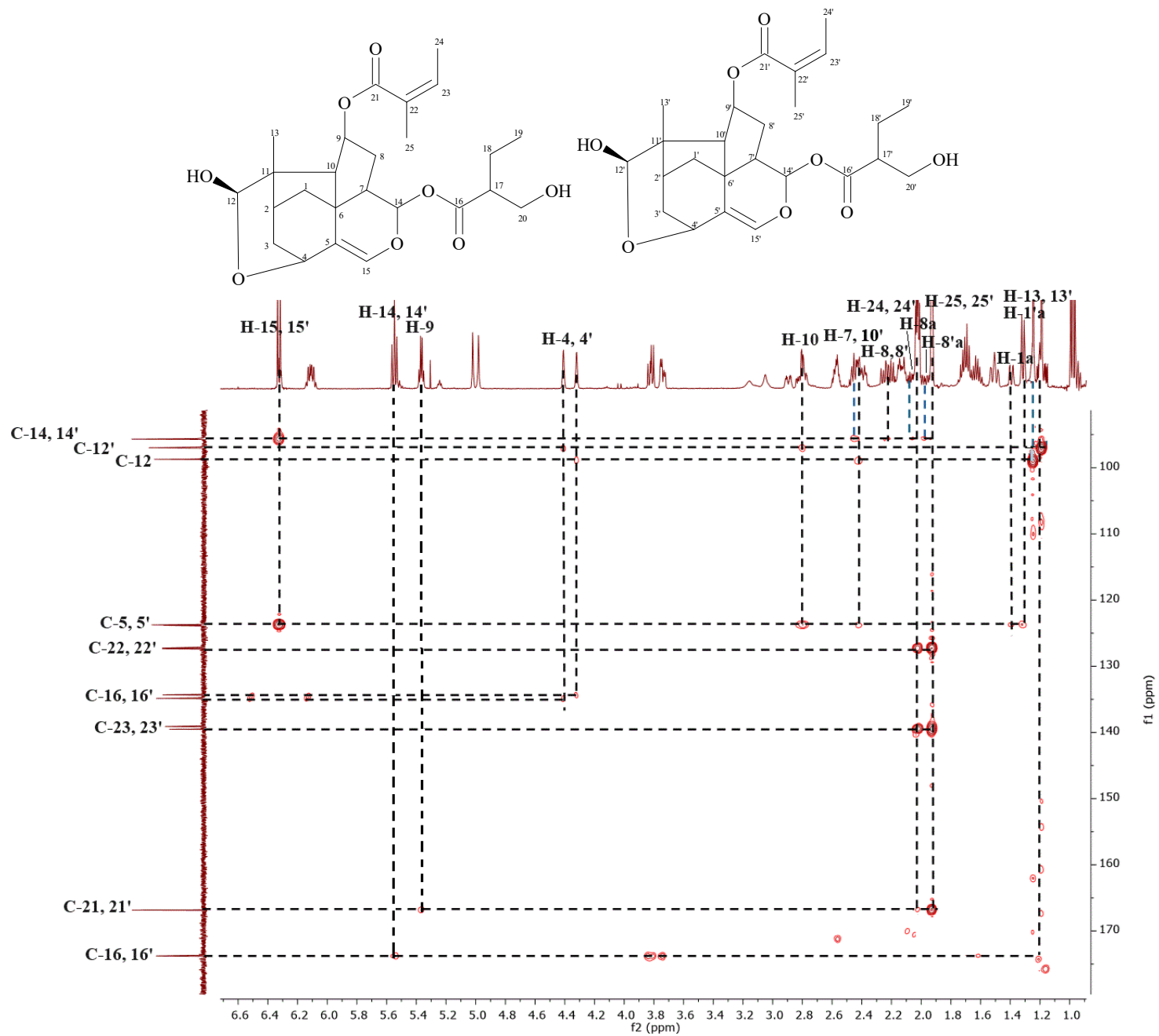

S6c Fig. HMBC spectrum of mixture of 1a and 1a' (500 MHz, CDCl<sub>3</sub>).

Supplement: S6c Fig — (PDF) [file pone.0339176.s010.pdf]

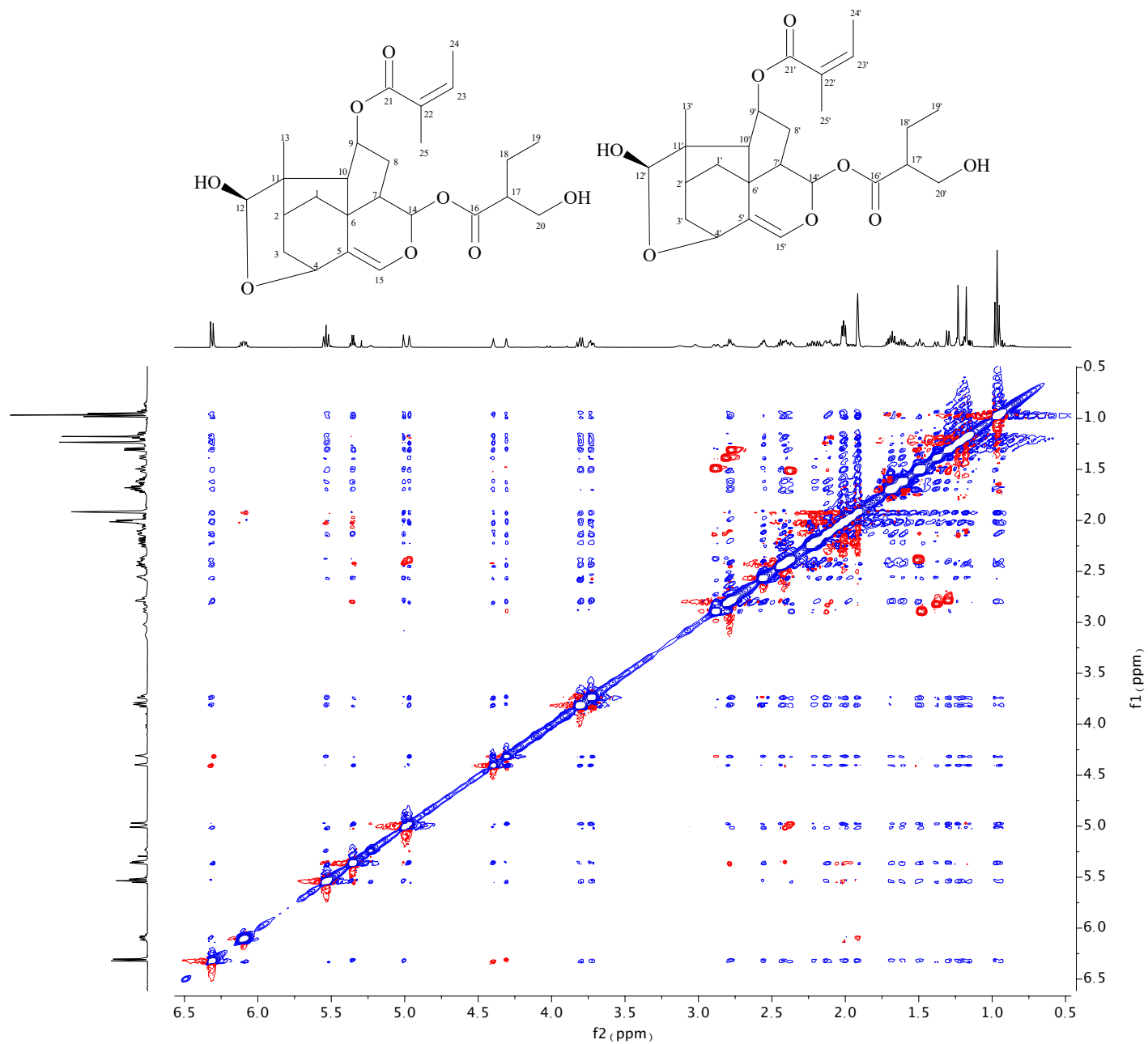

**S7 Fig. ROESY spectrum of mixture of 1a and 1a' (500 MHz, CDCl<sub>3</sub>).**

Supplement: S7 Fig — (PDF) [file pone.0339176.s011.pdf]
