# Supplementary material for: Trixis angustifolia DC. as a potential plant for the co-management of diabetes mellitus and tuberculosis
Source: PLoS One. 2025 Dec 31;20(12):e0339176. doi: 10.1371/journal.pone.0339176 (PMC12755760; doi:10.1371/journal.pone.0339176)
Supplement: S8 Fig — (PDF) [file pone.0339176.s012.pdf]

Display Report

Analysis Info

Analysis Name	D:\Data\Daniel\030916_ElenaQB.d	Acquisition Date	3/11/2016 10:59:17 AM
Method	tune_low.m	Operator	Daniel
Sample Name	030916_ElenaQB	Instrument / Ser#	micrOTOF-Q II 10392
Comment			

Acquisition Parameter

Source Type	ESI	Ion Polarity	Positive	Set Nebulizer	0.4 Bar
Focus	Active			Set Dry Heater	180 °C
Scan Begin	50 m/z	Set Capillary	4500 V	Set Dry Gas	4.0 l/min
Scan End	3000 m/z	Set End Plate Offset	-500 V	Set Divert Valve	Source

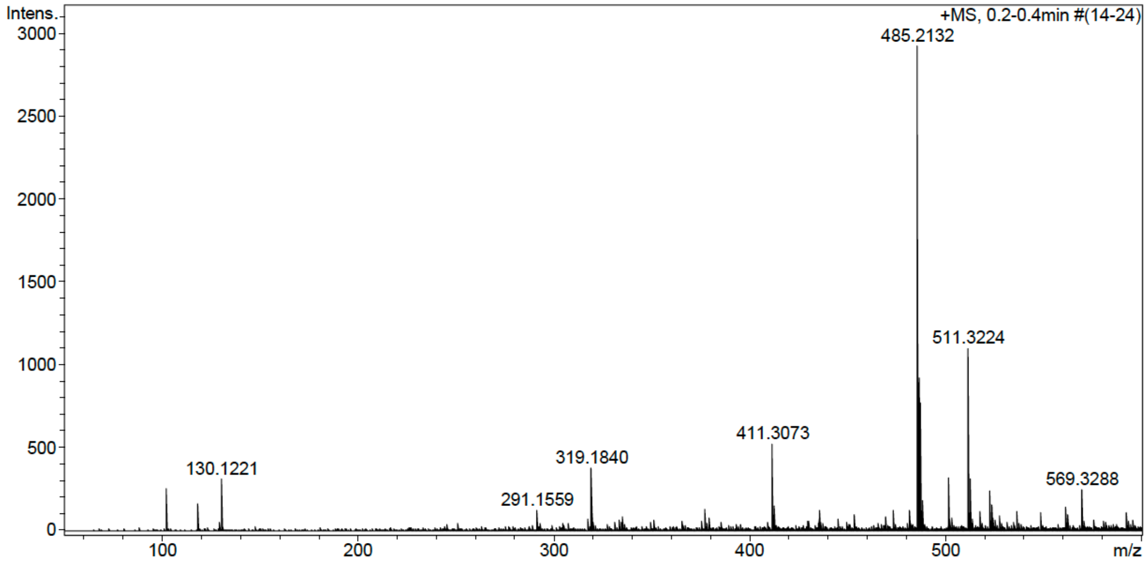

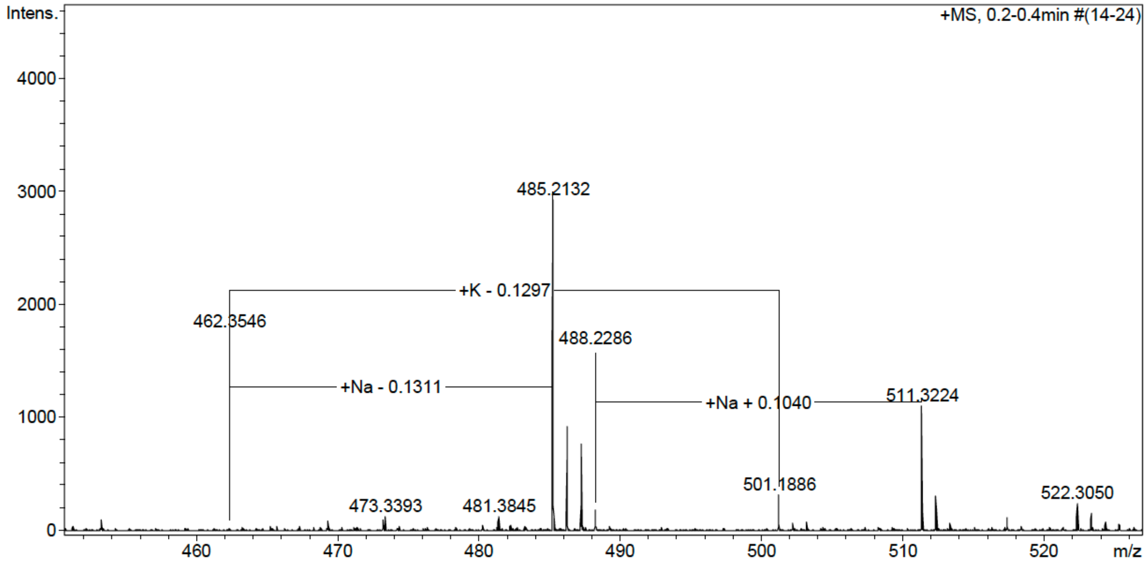

S8 Fig. HRMS-ESI spectrum (positive ion mode) of mixture of 1a and 1a'.
